# Lumenal components of cytoplasmic microtubules

**DOI:** 10.1042/BST20220851

**Published:** 2022-12-16

**Authors:** Chisato Tsuji, Mark P. Dodding

**Affiliations:** School of Biochemistry, Faculty of Life Sciences, University of Bristol, Bristol BS8 1TD, U.K.

**Keywords:** actin, cryo-EM, lumen, microtubule, tomography

## Abstract

The lumen of cytoplasmic microtubules is a poorly explored expanse of intracellular space. Although typically represented in textbooks as a hollow tube, studies over several decades have shown that the microtubule lumen is occupied by a range of morphologically diverse components. These are predominantly globular particles of varying sizes which appear to exist either in isolation, bind to the microtubule wall, or form discontinuous columns that extend through the lumenal space. Actin filaments with morphologies distinct from the canonical cytoplasmic forms have also now been found within the microtubule lumen. In this review, we examine the historic literature that observed these lumenal components in tissues from diverse species and integrate it with recent cryo-electron tomography studies that have begun to identify lumenal proteins. We consider their cell and tissue distribution, possible mechanisms of incorporation, and potential functions. It is likely that continuing work in this area will open a new frontier in cytoskeletal biology.

## Introduction

Microtubules are essential for a vast range of cellular processes, acting both as a dynamic adaptive mechanical scaffold and as a network for intracellular transport by dynein and kinesin family motors. Studies over many decades have uncovered the fundamental structural and biophysical properties of the cylindrical polymer, as well as a host of factors that control microtubule dynamics and function in diverse cellular contexts. With some exceptions, the field has generally focused on proteins that bind the exterior wall or the ends of microtubules; for example, molecular motors, microtubule associated proteins (MAPs), post-translational modifications, or proteins and protein complexes associated with nucleation or plus end dynamics [1–4] The textbook representation of the microtubule typically depicts it as a hollow, solvent filled, tube. However, over recent years, it has become clear that this representation requires revision; increasing evidence indicates that microtubules are not always hollow and can be occupied by a range of lumenal components. In studies of axonemal microtubules, a plethora of microtubule inner proteins (MIPs) have been identified which associate with the inner surface of the microtubule wall, and generally appear to be important for microtubule stability and structure. These studies have been reviewed elsewhere, with some very rapid recent progress, and so will not be considered in detail in this review [5–19]. Here, we consider the series of historic and recent studies that have focused on the lumenal contents of cytoplasmic microtubules which are less well understood. We discuss recent progress on the molecular identification and structural characterisation of these components in a range of cell types. We consider several models on how these proteins are incorporated into the microtubule and reflect on if and how they may impact on microtubule function and dynamics.

## Negative-stain electron microscopy of microtubules in animal tissues

Early evidence of a substantial lumenal component of cytoplasmic microtubules emerged from electron microscopy studies conducted from the 1960s to the 1990s. A series of papers explicitly reported and/or presented images showing a central ‘dot’ like density within the ≈15–17 nm diameter lumen [20] in transverse sections of microtubules from a diverse range of animal tissues. These included rat and human platelets [21–25], and neurons from human, rat [26–30], mouse [31], chicken [32], shrimp [33], *C. elegans* [34], frog [35,36], toad [37], and lamprey [38]. Further examples include insect cells from the blowfly [39] and locust [40]. Descriptions of the lumenal material found in these studies varied from granules to particles, to discontinuous columns, through to a central 4–6 nm diameter filament. Whether this reflected differences in sample preparation or bona-fide heterogeneity in composition was not clear. There was some concern that lumenal material could be an artefact of fixation and heavy metal staining in sample preparation. However, in the most substantive study from this era, Burton was able to observe the same material in cells without the use of osmium tetroxide heavy metal stain [35]. In most studies, lumenal density was present in some microtubules/sections but not others, and in the case of frog neurons, Burton observed more lumenal material in microtubules from the distal versus the proximal regions of axons [35], suggesting that the densities are not merely artefacts.

## Cryo-electron microscopy studies of cells and tissues

**Figure 1.**
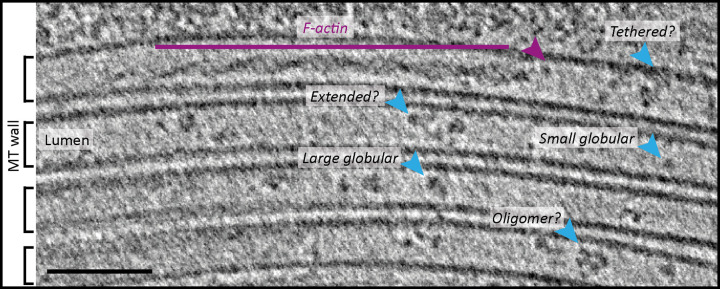
Morphologically diverse lumenal material in microtubules extruded from HAP1 cells. Tomogram subvolume showing a bundle of four adjacent microtubules within kinesore induced HAP1 cell projections. An actin filament resides within the uppermost microtubule lumen and potentially breaks off into G-actin (purple arrowhead). Globular densities are also visible in the lumen (blue arrows) which range from small and medium sized structures to more extended densities, some apparently associated with the microtubule wall. Figure is a new image acquired during the course of our work using the approach described in [50]. Scale bar is 50 nm.

Cryo-electron microscopy recapitulates the native structure of biological specimens more reliably than chemical fixation. This has enabled high-resolution imaging of the microtubule lumen not only in transverse section, but also along their length. Studies have confirmed the presence of heterogenous microtubule lumenal density in many different cell types. These include rat and mouse hippocampal neurons [41–46], neuronal cell lines [47], P19 murine embryonal carcinoma cells [41,48], HeLa cells [48], Ptk2 cells [49], HAP1 cells (Figure 1) [50], Drosophila neurons [43], mouse embryonic fibroblasts [51], human platelet filopodia [52], human cerebral organoids [53], as well as (using cryo-focused ion beam milling (cryo-FIB milling), *C. elegans* gut tissue [54] and Chinese hamster ovary (CHO) cells (using the cryo-electron microscopy of vitreous sections (CEMOVIS) approach) [55]. In general, descriptions of lumenal material have varied in size, morphology, and frequency, but together these historic and recent observations using a diverse range of electron microscopy approaches confirm that cytoplasmic microtubules are, in many biological contexts, not hollow.

## Morphological and structural characterisation of lumenal components

### Globular lumenal particles

In the first cryo-electron tomography (cryo-ET) study that explicitly aimed to characterise the lumenal densities, Garvalov et al. identified columns of globular particles of ∼7 nm diameter, enriched at depolymerising ends of microtubules in rat hippocampal neurons. These often appeared to be connected to the microtubule wall, at spacings of between 8–20 nm [41]. Similar distances and particle sizes were observed by Atherton et al. in mouse primary hippocampal neuron cultures [44,45]. Significant variation in the morphology of lumenal material has been observed in cryo-ET studies, with some studies reporting similar sized particles in a densely packed column [42,46], but others reporting a more varied distribution along the microtubule with particles of different sizes [48,51,53]. The location of particles within the lumen varies from the centre of the lumen (as observed in many of the negative stain images), to near the microtubule wall, including some with visible attachment to the wall through small projections. The frequency of the particles appears to differ depending on the cell type, cell regions and even between adjacent microtubules [41–43,45,46]. In general, evidence has varied as to where, and in which microtubules, lumenal particles may be enriched.

In a recent cryo-ET study of rat dorsal root ganglion axons, abundant globular lumenal particles (9 nm diameter, between 40 and 100 per micron of microtubule, sometimes tethered to the microtubule wall) were observed. The authors were able to use sub-tomogram averaging to yield a 32 Å structure showing that the particles are composed of several globular subdomains surrounding a central pore [43], providing important evidence that at least a subset of lumenal particles have a defined structure and composition. However, interestingly, in the same study, the authors noted that densities in *Drosophila* neuronal microtubules were smaller with a more variable size, shape, and distribution. Overall, these studies indicated that lumenal particles may come in several different forms of diverse composition that vary between species and/or cell type.

### Candidate protein components of lumenal particles

#### Microtubule associated protein 6 (MAP6)

Cuveillier et al. [56] reported the presence of lumenal densities in microtubules extracted from hippocampal neurons that were less abundant in samples from mice that lack MAP6 — a microtubule-associated protein associated with stability against cold and drug induced depolymerisation. Microtubules polymerised in the presence of MAP6 displayed lumenal densities of average 9 nm diameter and spaced at ∼31 nm intervals. The presence of lumenal MAP6 curves the microtubule into a long left-handed helix and leads to more breakages in the microtubule lattice. However, as noted by Foster et al. [43] MAP6 is predicted to be an unstructured protein and so is unlikely to be the sole constituent of the globular lumenal particles observed in previous studies.

#### Alpha tubulin acetyltransferase 1 (αTAT1)

αTAT1 acetylates α-tubulin at residue K40 which is located in the microtubule lumen [20]. K40 acetylation is associated with stable long-lived microtubules in cells [57–59]. αTAT1 can enter the lumen through the ends of microtubules, as well as through lattice defects *in vitro* [58,60,61]. It has been shown that αTAT1 can bind the exterior surface of microtubules [62] and is also transported along axons in neuronal vesicles by motor proteins and could be released at microtubule ends to promote acetylation [63]. The globular acetyltransferase domain is ∼5 nm in diameter [60,64], and so may be a component of at least some of the lumenal particles. Furthermore, as the major lumenal post-translational modification, it is worth considering whether acetylation could affect the incorporation or dynamics of other lumenal components. The *C. elegans* αTAT orthologues, MEC-17 and ATAT-2, have been shown to be important for the presence of lumenal material in touch receptor neurons although it is not clear whether their encoded proteins are a component of this material or this enables the recruitment of other proteins [65]. In the same study, tubulin acetylation was also shown to be important for controlling protofilament number, raising the question of whether protofilament number and dimensions of the lumen could also affect incorporation of lumenal material or vice versa.

#### Tau

Repeat motifs of Tau have been shown *in vitro* to bind the microtubule inner wall via β-tubulin [66]. Tau derived peptides are also able to promote the encapsulation of gold nanoparticles and GFP into the microtubule lumen which can increase microtubule stability [67–70]. Similar to MAP6, the mostly unstructured nature of Tau means that it is unlikely to account for the previously observed globular densities, but it is interesting to consider whether it may have a role in recruiting other proteins into the lumenal space.

#### Tubulin binding cofactors

Chakraborty et al. [71] recently reported sub-tomogram averaging on 7–10 nm particles found in the lumen of rodent primary hippocampal neurons, human iPSC derived neurons and murine pluripotent or differentiated P19 cells. Particles bound to the inner microtubule wall were found exclusively in the pluripotent P19 cells, whereas free particles were found in the lumen of all examined cell types. Fitting of this density to structural data in the EMDB/PDB, as well as Alphafold2 models suggested the possibility that tubulin binding cofactors are key components of several particles. The frequency of the particles differs depending on the cell type and cell differentiation state. There was also enhanced proximity to curved areas and broken areas of the microtubules, as well as plus ends of polymerising microtubules, suggesting a potential entry mechanism for the particles, and indicating that they may have a functional role in stabilising microtubules, or tubulin homeostasis.

#### Lumenal actin filaments

Most lumenal densities observed in cryo-ET studies appear to be globular. Recently however, using a small-molecule/kinesin driven extrusion technique to produce thin microtubule-based projections from HAP1 cells, Paul et al. [50] showed that F-actin can reside in the microtubule lumen in a cellular context. Microtubule lumenal F-actin was found in two distinct conformations, both with a higher degree of helical twist than the canonical muscle form. The first form, the Class I filament, had more similar helical properties (as judged from layer-line analysis) to cofilin-bound F-actin than the canonical form (strand crossovers at 29 nm for Class I filaments vs ≈27 nm for cofilactin vs ≈37 nm for muscle actin); the second form, the Class II filament, showed layer-line properties consistent with a novel actin conformation and/or the presence of actin binding proteins [50]. Interestingly, Class II filaments were associated with a slightly larger microtubule diameter, suggesting the possibility that either the typical 13 protofilament lattice is expanded or that there are more protofilaments. In some images of the microtubule lumenal actin (Figure 1), it is also possible to see apparent dissociation of the actin at the ends of the filament. This suggests the possibility that some of the globular densities that have been observed in other studies could be G-actin or low order actin oligomers. If so, this requires a reconsideration of actin dynamics, where the protomer is present at high local concentration and in a unique confined environment, where a classic diffusion-driven treadmilling framework may not apply.

## Mechanisms of entry into the microtubule lumen

**Figure 2.**
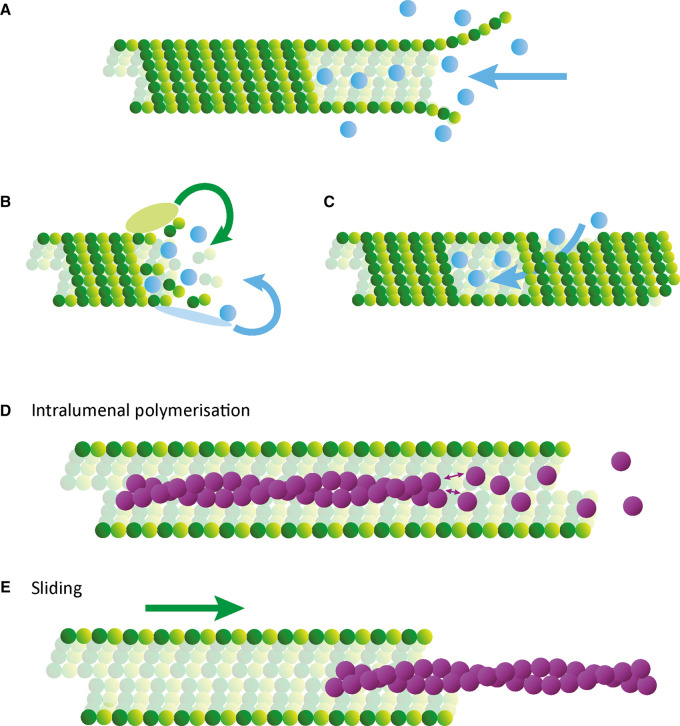
Diagram to illustrate potential mechanisms of protein entry into the microtubule lumen. Globular proteins could enter in three ways: (**A**) encapsulation from growing or stable ends of microtubules, (**B**) active incorporation during microtubule polymerisation, or (**C**) entry through microtubule breakages. (**D**) Actin filaments could polymerise within the microtubule from free G-actin. (**E**) F-actin entry could be driven by sliding motions of microtubules.

Robust identification of lumenal components in cytoplasmic microtubules must be a priority going forwards. This should proceed in parallel with consideration of how they are incorporated into the dynamic microtubule (Figure 2). Some densities have been reported to have a higher residency near ends of microtubules [25,41,46] or near breakages, whereas in some cases there have been no significant correlation with these sites [43,45]. Conceptually, there are three possibilities of entry that are not mutually exclusive. First, a polymerising microtubule may engulf cytosolic proteins. One could envisage this as a rather non-specific passive process; alternatively, a more specific mechanism driven by affinity for the lumenal wall or MAPs at the growing tip, or possibly, targeted release from vesicles at microtubule ends after it has been transported along the microtubule by motor proteins. Indeed, microtubule polymerisation is required for the lumenal entry of MAP6 *in vitro* [56]. Second, proteins could enter the lumen through open microtubule ends; this could again be facilitated by microtubule end associated proteins and/or affinity for the lumenal wall, or by microtubule sliding motions (Figure 2A,B). However, it is worth noting that the diffusion of any particles with affinity for the microtubule lumen is likely to be very slow [72]. Finally, proteins could actively or passively enter the lumen through breaks in the lattice (Figure 2C). For the large globular densities discussed above, it is difficult to envisage an entirely diffusion-driven process and reports of connections to the microtubule wall suggest that interactions with tubulin could help to drive incorporation as the microtubule is being polymerised. It is worth noting that *in vitro*, antibodies against acetylated K40 can enter the lumen [57,61]. Fluorescently labelled FKBP (FK506 binding protein) can also be targeted to FRB (FKBP-rapamycin binding domain of mTOR) fused to the lumenal side of tubulin in a rapamycin dependent manner; this appears to occur through a combination of incorporation through polymerisation and diffusion through ends and breakages [73]. The entry mechanism likely differs depending on the size of particles and affinity to tubulin. The microtubule properties *in vivo* versus *in vitro*, as well as fixation methods which affect microtubule integrity, must be considered as well when interpreting experiments.

Compared with globular proteins or protein complexes, F-actin poses a different challenge. Polymerisation of tubulin around an existing actin filament seems plausible, as does the microtubule-sliding driven insertion into an open microtubule end or a break in the lattice (Figure 2D,E). In the system used by Paul et al. [50], microtubules are actively sliding, and it is likely that they are under a high degree of structural and mechanical stress. This may also be the case in platelets where motor driven microtubule sliding plays a key role in their dynamics [74]. Actin incorporation may be favoured by the presence of specific actin binding proteins. Indeed, both MAP6 and Tau contain actin binding domains [75,76]. Alternatively, G-actin could be incorporated into the growing microtubule at or near its cytosolic concentration. It is noteworthy that the cytosolic concentration of G-actin is well-above the critical concentration for polymerisation, and in the absence of the complex cytosolic buffering system (profilin/β-thymosin), F-actin could spontaneously polymerise within the lumen. There may be an interesting analogy to be drawn in the growth of gold nanowires within the microtubule lumen through targeting of gold nanoparticle seeds using antibody fragments directed at acetylated K40 [77]. Finally, it is also interesting to consider a co-nucleation/polymerisation model; the structure of the microtubule nucleating complex, γ-tubulin ring complex, revealed an actin-like protein as a core-component, although this appears to be in the wrong orientation to nucleate actin at the same time unless a major conformational change takes place [78–81].

## Towards a function for lumenal components

The high abundance and differences in the frequency of microtubule lumenal contents between adjacent microtubules, regions within a cell and between different types of cells suggest that the filaments and particles are not present by simple coincidence, but may have specific functions. A significant proportion of lumenal densities have been identified in neurons, particularly within axons, which require very stable microtubules that extend for long distances in a confined environment; although this may reflect a bias in the types of sample that have been extensively studied rather than a physiological difference. Lumenal particles or actin filaments could be providing a stabilising effect on microtubules through distinct mechanisms. Encapsulation of GFP within the lumen using a tau derived peptide, that binds directly to the microtubule wall, has been shown to stabilise microtubules and increase their rigidity *in vitro* [82] and lumenal MAP6 *in vitro* has also been shown to increase microtubule stability [56]. Binding of proteins on the inner surface of the microtubule may be a general mechanism to modulate microtubule dynamics (to stabilise or destabilise) from the inside without restricting the binding of other proteins on what is presumably a quite congested outer surface. The possibility of tubulin cofactors residing within the lumen may also point to a mechanism for localised microtubule repair [71]. One could consider whether content flow could occur inside the lumen. The idea of the microtubule lumen being used for transport was proposed by Slautterback [83] and has been alluded to several times since. However, even if facilitated by binding to the microtubule lumen, the movement of the proteins within the lumen is likely to be very slow, compared with the speed of transport using motor proteins on the exterior surface.

At this point, it is perhaps worth returning to studies of axonemal microtubules where structural and functional understanding is more advanced [5–16]. Numerous MIPs, both globular and filamentous, form a periodic mesh lining the inner wall of the microtubule, some of which protrude into the microtubule wall itself, to stabilise the microtubule in cilia and sperm flagella. At least at this point, this periodic highly ordered assembly of well-defined proteins stands in contrast with the looser and more heterogenous material observed for cytoplasmic microtubules.

## Summary

**Figure 3.**
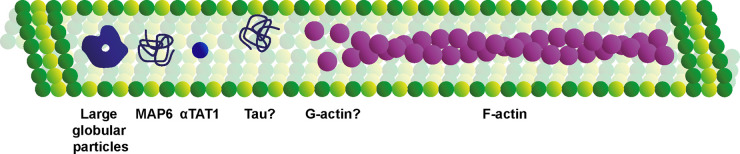
Summary of lumenal components described in this review. Both candidate and experimentally demonstrated proteins include a variety of large globular particles of unclear composition, the unstructured proteins MAP6 and Tau, the small globular protein αTAT1, as well as G- and F-actin.

Imaging by both chemical fixation and modern cryo-ET have shown that the microtubule lumen is not always hollow as is generally represented (Figure 3). The majority of observed densities have been in neurons, which could reflect their function, but also the thinner, more accessible nature of neurons for imaging. The development of techniques such as CEMOVIS and cryo-FIB milling have enabled studies of microtubules in thicker tissues [41,54,55] and will allow us to continue to look deeper into cells and broaden our understanding of where lumenal densities are present. Further structural understanding using cryo-ET and subvolume averaging is required for their identification; this should also help reveal the entry mechanisms and their function, as well as resolve outstanding questions on whether lumenal components are associated with particular microtubule architectures (e.g. protofilament number). This will come with significant challenges – most notably how to specifically perturb and assess the function of a lumenal component without affecting roles that they might have on the exterior of microtubules or in the cytosol. As such, *in vitro* reconstitution approaches to understand the effect of microtubule lumenal proteins on microtubule structure and dynamics will be particularly important. This may allow the definition of mutations that uncouple cytosolic and lumenal activities. When these are combined with rigorous *in situ* studies, we will begin to understand the function of microtubule lumenal components in a complex cellular context.

## Perspectives

- Observation of proteins in the microtubule lumen in both negative-stain and cryo-electron microscopy studies challenges the classic representation of cytoplasmic microtubules as a hollow cylindrical polymer.
- Recent studies show significant heterogeneity in the morphology, distribution, and frequency of particles in the cytoplasmic microtubule lumen.
- Both *in vitro* and *in vivo* approaches must be integrated to identify particles and enable their manipulation, specifically in the microtubule lumen, to understand their entry mechanisms and function in the context of the dynamic and complex cell environment.

## Open Access

Open access for this article was enabled by the participation of University of Bristol in an all-inclusive *Read & Publish* agreement with Portland Press and the Biochemical Society under a transformative agreement with JISC.

## Abbreviations

CEMOVIS: cryo-electron microscopy of vitreous sections
MAPs: microtubule associated proteins
MIPs: microtubule inner proteins
